# Rates of COVID-19–Related Outcomes in Cancer Compared With Noncancer Patients

**DOI:** 10.1093/jncics/pkaa120

**Published:** 2021-01-21

**Authors:** Lova Sun, Sanjna Surya, Anh N Le, Heena Desai, Abigail Doucette, Peter Gabriel, Marylyn D Ritchie, Daniel Rader, Ivan Maillard, Erin Bange, Alexander C Huang, Robert H Vonderheide, Angela DeMichele, Anurag Verma, Ronac Mamtani, Kara N Maxwell

**Affiliations:** 1 Division of Hematology/Oncology, Department of Medicine, Perelman School of Medicine, University of Pennsylvania, Philadelphia, PA, USA; 2 Department of Radiation Oncology, University of Pennsylvania, Philadelphia, PA, USA; 3 Abramson Cancer Center, University of Pennsylvania, Philadelphia, PA, USA; 4 Department of Genetics, Perelman School of Medicine, University of Pennsylvania, Philadelphia, PA, USA

## Abstract

Cancer patients are a vulnerable population postulated to be at higher risk for severe coronavirus disease 2019 (COVID-19) infection. Increased COVID-19 morbidity and mortality in cancer patients may be attributable to age, comorbidities, smoking, health care exposure, and cancer treatments, and partially to the cancer itself. Most studies to date have focused on hospitalized patients with severe COVID-19, thereby limiting the generalizability and interpretability of the association between cancer and COVID-19 severity. We compared outcomes of SARS-CoV-2 infection in 323 patients enrolled in a population-based study before the pandemic (n = 67 cancer patients; n = 256 noncancer patients). After adjusting for demographics, smoking status, and comorbidities, a diagnosis of cancer was independently associated with higher odds of hospitalization (odds ratio = 2.16, 95% confidence interval = 1.12 to 4.18) and 30-day mortality (odds ratio = 5.67, 95% confidence interval = 1.49 to 21.59). These associations were primarily driven by patients with active cancer. These results emphasize the critical importance of preventing SARS-CoV-2 exposure and mitigating infection in cancer patients.

Cancer patients have a higher risk of coronavirus disease 2019 (COVID-19) complications than the general population (1-10) due in part to factors such as older age, higher smoking rates, comorbidities, frequent health care exposures, and effects of cancer therapies. Hospitalized cancer patients have been shown to have higher rates of severe COVID-19 compared with noncancer controls matched for age (4), sex, and comorbidities (11). Recent administration of anticancer therapies has been associated with higher risk of mortality or complications from SARS-CoV-2 (4,6,10-12). Because most studies have focused on cancer patients hospitalized with severe COVID-19, it is unclear whether cancer status has an independent adverse impact on clinical outcomes in a population-based group of patients diagnosed with SARS-CoV-2 infection. We leveraged the Penn Medicine BioBank (PMBB), an institutional review board–approved, population-based cohort allowing access to electronic health record (EHR) data (13) to investigate the association between cancer status and COVID-19 outcomes.

Patients who had consented before the COVID-19 pandemic to enrollment in PMBB under a University of Pennsylvania institutional review board–approved protocol, and were subsequently found by symptom-driven or preprocedural clinical testing (Supplementary Table 1, available online) to have SARS-CoV-2 infection by reverse transcriptase polymerase chain reaction, were identified from the Penn EHR. Cancer patients met at least 1 of 3 criteria: 1) 3 or more International Classification of Diseases 10th Revision (ICD-10) billing codes for an invasive cancer, 2) inclusion in the Penn Medicine Cancer Registry, and 3) 1 visit within a Cancer Service Line clinic. Cancer diagnoses were confirmed by manual chart review. Patient characteristics and clinical outcomes were extracted from the EHR and compared between patients with and without cancer. Separate multivariable logistic regressions were performed to estimate odds ratios (OR) and 95% confidence intervals (CI) for the association between cancer diagnosis and COVID-19 outcomes (hospitalization, intensive care unit [ICU] admission, and mortality in the 30 days following COVID-19 diagnosis) adjusted for potential confounders, including demographic factors, smoking status, comorbidities, and socioeconomic status estimated by national poverty index based on neighborhood mapping (14). Exploratory subgroup analyses were performed to investigate these associations among patients with active cancer (defined as having metastatic disease and/or receiving cancer-directed systemic therapy, radiation therapy, or surgical resection in the 2 months before COVID-19 diagnosis) (15) compared with noncancer patients as well as those with cancer in remission compared with noncancer patients. Statistical analyses were performed using a *t* test for continuous variables and χ^2^ test for categorical variables; statistical significance was defined as a 2-sided *P* less than .05.

As of June 2020, of 4816 patients previously enrolled in PMBB who had been tested for COVID-19, 323 (7.3%) had laboratory-confirmed SARS-CoV-2 infection. Of COVID-19–positive patients, 67 (20.7%) had a cancer diagnosis (80.6% with solid tumor malignancy; 26.9% with active cancer). Compared with noncancer patients, COVID-19–positive cancer patients were more likely to be older (62 vs 50 years, *P* < .001), male (53.7% vs 39.5%, *P* = .04), and have a history of smoking (55.2% vs 35.0%, *P* = .003; Table 1). Notably, the proportion of Black patients was statistically significantly higher in both cancer and noncancer COVID-19–positive patients (65.7% and 64.1%, respectively) compared with all PMBB patients tested for SARS-CoV-2 (32.0%; for both, *P* < .001).

**Table 1.** Baseline characteristics of cancer vs noncancer COVID-19–positive patients

Characteristic	Cancer patients (n = 67)	Noncancer patients (n = 256)	*P*
Median age (interquartile range), y	62 (53-71)	50 (37-60)	<.001^a^
Race, No. (%)			
White	18 (26.9	74 (28.9)	.98^b^
Black	44 (65.7)	164 (64.1)	
Asian	2 (3.0)	5 (2.0)	
Other	2 (3.0)	8 (3.1)	
Unknown	1 (1.5)	5 (2.0)	
Sex, No. (%)			
Female	31 (46.3)	155 (60.5)	.04^b^
Male	36 (53.7)	101 (39.5)	
Ever smoker, No. (%)	37 (55.2)	90 (35.2)	.003^b^
Mean national poverty percentile (range)	91 (63-93)	91 (76.5-93)	.29^a^
Comorbidities, No. (%)			
Hypertension	38 (56.7)	117 (45.7)	.11^b^
Diabetes	22 (32.8)	72 (28.1)	.45^b^
Obesity	12 (17.9)	76 (29.7)	.05^b^
Pulmonary disease	14 (20.9)	36 (14.1)	.17^b^
Mood disorders	8 (11.9)	37 (14.5)	.60^b^
Ischemic cardiovascular disease	11 (16.4)	29 (11.3)	.26^b^
Immunodeficiency	5 (7.5)	11 (4.3)	.29^b^
Cerebrovascular disease	3 (4.5)	8 (3.1)	.59^b^

a Two-sided *P* values calculated using Wilcoxon rank sum test.

b Two-sided *P* values calculated using Pearson’s χ^2^ test.

Rates of hospitalization, ICU admission, and 30-day mortality were higher in patients with cancer compared with those without cancer (55.2% vs 28.9%, 25.4% vs 11.7%, and 13.4% vs 1.6%, respectively) (Table 2). Older age, Black race, and number of comorbidities were statistically significantly associated with increased odds of hospitalization and ICU admission (all *P* < .05). In fully adjusted models, cancer diagnosis was associated with statistically significantly increased odds of hospitalization (OR = 2.16, 95% CI = 1.12 to 4.18) and 30-day mortality (OR = 5.67, 95% CI = 1.49 to 21.59), but not ICU admission (OR = 1.91, 95% CI = 0.90 to 4.06). In exploratory subgroup analyses by cancer status (ie, active vs remission), adjusted associations with hospitalization, ICU admission, and 30-day mortality were strongest in the subgroup of patients with active cancer compared with noncancer patients (data not shown), suggesting that the association between cancer diagnosis and poor COVID-19 outcomes was driven primarily by patients with active cancer.

**Table 2.** Event rates and odds ratios of clinical outcome in cancer vs noncancer COVID-19–positive patients

Outcome	Patients with cancer	Patients without cancer
Hospitalization		
Event rate, %	55.2	28.9
Unadjusted OR (95% CI)	3.03 (1.75 to 5.27)	Ref
Adjusted OR^a^ (95% CI)	2.16 (1.12 to 4.18)	Ref
ICU Admission		
Event rate, %	25.4	11.7
Unadjusted OR (95% CI)	2.56 (1.31 to 5.00)	Ref
Adjusted OR^a^ (95% CI)	1.91 (0.90 to 4.06)	Ref
30-Day mortality		
Event rate, %	13.4	1.6
Unadjusted OR (95% CI)	9.78 (2.91 to 32.85)	Ref
Adjusted OR^a^ (95% CI)	5.67 (1.49 to 21.59)	Ref

a Adjusted for age, race, gender, socioeconomic status, smoking, comorbidities. CI = confidence interval; ICU = intensive care unit; OR = odds ratio; Ref = reference population.

Concerns about risk of COVID-19 in cancer patients have led to alterations in cancer care such as treatment regimen modifications, delayed screening, decreased clinical trial enrollment, and increased telemedicine use (16)—the potential effects of which on cancer outcomes are as of yet unknown, although models predict a negative impact on survival (17). Prior studies investigating the association between cancer and COVID-19 outcomes have largely focused on hospitalized patients with severe disease who may not be representative of the general population. In particular, studies utilizing nonrandom samples, of which hospitalized patients are a classic example, are prone to collider bias, which can induce spurious or distorted associations (18). Though other studies have included nonhospitalized patients (12,15), our cohort of COVID-19–positive patients was identified from a large population-based cohort defined before the pandemic, which allowed more unbiased sampling of cancer and noncancer patients. Our finding that cancer patients with COVID-19 were more likely than noncancer patients to experience hospitalization and death even after adjusting for patient-level factors supports the hypothesis that cancer is an independent risk factor for poor COVID-19 outcomes. In addition, poorer outcomes seen in Black patients within our cohort parallel prior reports showing the disproportionate impact of COVID-19 on minority communities (19).

Our study had several limitations. Our sample size precluded subgroup analysis of different cancer types, which may confer nonuniform risk of severe COVID-19 (1,3,10). Similarly, our cohort contained a relatively small number of patients with active cancer, which precluded analysis of the impact of different cancer therapies on COVID-19 outcomes. A prospective observational study of patients in a United Kingdom cancer center network did not find an association between cancer therapy type and mortality (15). Given the conflicting evidence on the impacts of active vs nonactive cancer status, cancer stage, and cancer therapies on COVID-19 prognosis, deeper investigation of these variables is needed (2-4,10,12,15).

In conclusion, in our cohort of COVID-19–positive patients identified from a health system population–based academic cohort, a diagnosis of cancer was strongly and independently associated with poor COVID-19 clinical outcomes, including hospitalization and 30-day mortality. Given that it is critically important that these patients engage with the health care system for optimal cancer-directed management, our results suggest that patients with cancer, particularly those receiving active treatment, should be among groups specifically targeted for COVID-19 mitigation and prevention strategies such as vaccination.

## Funding

This work was supported by the National Institutes of Health (P30-CA016520 to Abramson Cancer Center).

## Notes

**Role of the funder:** The funder had no role in the design of the study; the collection, analysis, and interpretation of the data; the writing of the manuscript; and the decision to submit the manuscript for publication.

**Disclosures:** Dr. Vonderheide reports having received consulting fees or honoraria from Celldex, Lilly, Medimmune, and Verastem; and research funding from Apexigen, Fibrogen, Inovio, Janssen, and Lilly. He is an inventor on a licensed patent relating to cancer cellular immunotherapy and receives royalties from Children’s Hospital Boston for a licensed research-only monoclonal antibody. Other authors declare that they have no competing interests.

**Prior presentations:** This manuscript has not been presented publicly. The manuscript was submitted online to medRxiv, and can be found under doi: https://doi.org/10.1101/2020.08.14.20174961.

**Acknowledgments:** We acknowledge the Penn Medicine BioBank (PMBB) for providing data and thank the patient-participants of Penn Medicine who consented to participate in this research program. The PMBB is approved under IRB protocol# 813913 and supported by Perelman School of Medicine at University of Pennsylvania.

**Author contributions:** Conceptualization (KNM, RM, AV, ADM, RHV, AH, IM, LS); Data curation (LS, SS, ANL, HD, AD, PG, MR, DR, AV); Formal Analysis (LS, AV, RM, KNM); Funding acquisition (RHV, ADM, RM, KNM); Investigation (AD, PG, MR, DR, AV); Methodology (LS, AV, RM, KNM); Project administration (LS, EB); Resources (AD, PG, MR, DR, AV, RHV); Software (AD, PG, AV, MR); Supervision (IM, A, RHV, ADM, AV, RM, KNM); Validation (LS, HD); Visualization (LS); Writing—original draft (LS, ADM, AV, RM, KNM); Writing—review and editing (all authors).

## Data Availability

The data underlying this article will be shared on reasonable request to the corresponding author.

## Supplementary Material

pkaa120_Supplementary_Data

## Notes

These authors equally contributed to this manuscript.
