# Supplementary material for: Rates of COVID-19–Related Outcomes in Cancer Compared With Noncancer Patients
Source: JNCI Cancer Spectr. 2021 Jan 21;5(1):pkaa120. doi: 10.1093/jncics/pkaa120 (PMC7853171; doi:10.1093/jncics/pkaa120)
Supplement: pkaa120_Supplementary_Data [file pkaa120_supplementary_data.pdf]

Supplementary Table 1. Diagnosis Codes Associated with Positive SARS-CoV-2 Polymerase Chain Reaction Tests (N=323)

Indication for testing	No. of patients (%)
Respiratory symptom or suspected COVID-19	163 (71.3)
Fever or chills	8 (3.5)
Loss of taste or smell	5 (2.2)
Preoperative or screening test	7 (3.1)
Unknown or other[a]	46 (20.1)

[a]Patients tested at drive-through testing centers did not have diagnosis codes associated with SARS-CoV-2 Polymerase Chain Reaction Tests
